# Supplementary material for: Creation of a Pilot School Health Research Network in an English Education Infrastructure to Improve Adolescent Health and Well-Being: A Study Protocol
Source: Int J Environ Res Public Health. 2022 Oct 21;19(20):13711. doi: 10.3390/ijerph192013711 (PMC9603152; doi:10.3390/ijerph192013711)
Supplement: Supplementary file 1 [file ijerph-19-13711-s001.zip › ijerph-1878074-supplementary.pdf]

# South West School Health Research Network Logic Model

## TARGET
(Who is intervention for?)

### Individuals
- Students in Year 8
- Students in Year 10
- Secondary school staff

### Stakeholder Groups
- Directors of Public Health
- Directors of Children's Services
- Directors of Education
- School senior leadership teams

### Systems
- Individual schools
- Academy/multi-academy trusts
- Local authorities

## SW-SHRN INTERVENTION
(What is the intervention?)

### Data Collection
- Student survey every 2 years
- School environment survey
- Stakeholder interviews
- Health economic data
- Linking with routine health and education datasets

### Reporting Results to Participants
- Benchmarked reports for schools
- Benchmarked reports for local authorities
- Feedback meeting with schools

### Working with Participants
- Health promotion worker
- Network-wide events and resources

### Wider Dissemination and Engagement
- Network website and newsletters
- Public events and webinars

## CHANGE MECHANISMS
(How & why does the intervention work?)

### Identification
- Target health and wellbeing domains requiring intervention
- Current health policies and practice in schools

### Knowledge Mobilisation
- Share evidence base with schools and local authorities
- Knowledge sharing across schools and facilitation of partnership working

### Tracking Over Time
- Monitor change and impact of interventions or policies
- Explore health trajectories over time
- Relationships between health and wellbeing domains and behaviours
- Relationships between school culture, environment, and practice, and health and wellbeing outcomes

## OUTCOMES
(What difference will it make?)

### Student Outcomes
- Improved health & wellbeing *(assessed via student survey)*
- Reduction in health risk behaviours *(assessed via student survey)*
- Improved educational attainment *(assessed via future data linkage)*

### School Outcomes
- Improvements in key student outcomes (assessed via future data linkage)
- Improved understanding of student health and wellbeing (assessed via student & school environment survey)
- Improved knowledge of evidenced-based health interventions policies and more effective policy making (assessed via school environment survey)
- Improved partnership working (assessed via qualitative interviews & network events)
- Improved cost effectiveness of health spending (assessed via health economic evaluation)

### Wider Outcomes
- More efficient use of health funding (assessed via health economic evaluation)

## MODERATORS (What factors will influence the change process?)

### School Level
- School IT access for online survey
- School culture
- School status (e.g. academy)
- School demographics
- School/Senior leadership buy-in
- Relationship between partners involved
- Knowledge exchange between members

### Network Level
- Number of schools involved
- Diversity of schools involved

### Wider Context
- Local authority buy-in
- Existing health and wellbeing surveys
- Curriculum pressures

**15 local authorities in the South West of England**

| Local Authority Name |
|---|
| Bath and North East Somerset |
| Bournemouth, Christchurch and Poole |
| Bristol |
| Cornwall |
| Devon |
| Dorset |
| Gloucestershire |
| Isles of Scilly |
| North Somerset |
| Plymouth |
| Somerset |
| South Gloucestershire |
| Swindon |
| Torbay |
| Wiltshire |
